# An eight-season analysis of the teams’ performance in the Spanish LaLiga according to the final league ranking

**DOI:** 10.1371/journal.pone.0299242

**Published:** 2024-02-28

**Authors:** Ibai Errekagorri, Javier Fernandez-Navarro, Roberto López-Del Campo, Ricardo Resta, Julen Castellano

**Affiliations:** 1 Society, Sports and Physical Exercise Research Group (GIKAFIT), Department of Physical Education and Sport, Faculty of Education and Sport, University of the Basque Country (UPV/EHU), Vitoria-Gasteiz, Spain; 2 The Football Exchange, Research Institute for Sport and Exercise Sciences, Liverpool John Moores University, Liverpool, United Kingdom; 3 Department of Competitions and Mediacoach, LaLiga, Madrid, Spain; University of Montenegro, MONTENEGRO

## Abstract

This study aimed to analyse the performance of 5,518 collective observations of the Spanish *LaLiga* teams for eight consecutive seasons (from 2011–12 to 2018–19), considering the final league ranking. The teams were divided into four groups: Europe (from 1^st^ to 6^th^), Upper-Middle (from 7^th^ to 11^th^), Lower-Middle (from 12^th^ to 17^th^) and Relegation (from 18^th^ to 20^th^). The variables recorded were: Passes, Successful Passes, Crosses, Shots, Goals, Corners, Fouls, Width, Length, Height, distance from the goalkeeper to the nearest defender (GkDef), total distance covered (TD) and number of points accumulated. The main results were that: 1) Europe, being superior to the rest of the groups, showed lower values of Length from 2015–16, and lower values of GkDef from 2014–15; 2) Upper-Middle showed lower values of Length from 2015–16; 3) Lower-Middle showed fewer Shots from 2013–14, and lower values of Length, GkDef and TD from 2014–15; and, 4) Relegation barely showed significant differences between seasons in any variable. The study concludes that the teams of the Europe, Upper-Middle and Relegation groups showed quite stable performance, while the teams of the Lower-Middle group presented a worsening in different dimensions as the seasons progressed. The information provided in this study makes it possible to have reference values that have characterized the performance of the teams for each group.

## Introduction

With the development of technology in sports and particularly in football, it has been possible to carry out more precise and objective studies about the performance of football players and teams during competition [1]. Nowadays, tracking systems (e.g., global navigation satellite systems or global positioning systems, local positioning systems, and semi-automatic video cameras) allow the analysis of kinematic variables (e.g., displacements, accelerations), as well as individual (e.g., heat maps) and collective (e.g., average positioning of the players) tactical variables of a team (e.g., distances between players and/or spaces covered by a group of players) based on the recorded positioning data [2–4]. The use of variables measuring physical and tactical aspects and covering individual player and teams’ units is essential to evaluate the performance of players and teams in competition [5], and even to carry out longitudinal monitoring.

Previous studies explored the development of the game of football throughout the years [6–13]. Considering this longitudinal viewpoint, several studies have focused on analysing physical aspects [6, 7, 9, 10, 12]. In this regard, previous studies analysed the evolution of the English *Premier League* teams throughout seven seasons [7], considering the specific position of players [10] or the final ranking of teams at the end of the season [9]. Barnes et al. [7] reported that the distance covered by the teams in the English *Premier League* had not changed much throughout the seven years, this way increasing the number of high-intensity actions and accumulated distance, as well as the number of sprints and accumulated distance. Bradley et al. [9] showed that all the English *Premier League* teams increased the high-intensity distance covered when they were not in possession of the ball throughout the seven seasons. However, teams that finished fifth to eighth by the end of the season showed a slight increase in the short distance covered in high intensity when in possession of the ball compared to other teams. The teams ranked fifth to eighth also showed a significant increase in the distance covered while sprinting compared to other teams. Regarding the Spanish *LaLiga*, a recent study [12] showed a small decrease in the total distance covered by the teams throughout eight seasons. However, the Spanish *LaLiga* teams performed a higher number of high-intensity efforts as the seasons progressed, and the Upper-Middle ranked teams (from 6^th^ to 10^th^) and Lower ranked teams (from 16^th^ to 20^th^) covered a greater distance at high-intensity [12].

Nevertheless, the technical-tactical dimension has also received considerable attention in the scientific literature [7–13]. Thus, Barreira et al. [8] observed and recorded 45 matches and 6,791 attacks in the semi-finals and finals of the *UEFA Euro Championship* and the *FIFA World Cup* from 1982 to 2010. They concluded that similar attacks led by top-tier football teams had moved away from a more individualised behaviour, such as dribbling and feints in the centre of the pitch, to a more group-based performance, such as short passes and crosses into the box. Wallace & Norton [13] analysed the evolution of game-play in international competitions (*FIFA World Cups*) throughout a 44-year period. These researchers indicated that the speed of football had increased due to a significant boost in the number of passes in the last few years. As for domestic leagues, there has been an increase in the number of passes and their effectiveness in the English *Premier League* over seven seasons, mainly short and medium-distance passes [7]. During the seven-season period analysed in the study, the Tier A teams (from 1^st^ to 4^th^) in the English *Premier League* demonstrated the greatest number of technical events and the highest levels of technical performance (i.e., number of passes and successful passes) [9]. However, the greatest increases in the technical parameters of passes made and received were shown by the Tier B teams (from 5^th^ to 8^th^). On the other hand, a recent study [12] found that the Spanish *LaLiga* technical performance evolution throughout an eight-season period is dependent on the level of the teams. Top (from 1^st^ to 5^th^), Upper-Middle (from 6^th^ to 10^th^), and Lower-Middle (from 11^th^ to 15^th^) ranked teams showed the greatest changes in different technical parameters as the seasons progressed (e.g., fewer shots, tackles or clearances, and more short passes, long passes, or aerial duels). On the contrary, Lower ranked (from 16^th^ to 20^th^) teams showed more stable technical performance.

Nevertheless, it could be interesting to have more information about the evolution of the teams’ performance in the Spanish men’s top professional football division according to the final league ranking, especially the evolution of the teams’ technical-tactical and physical performance [14]. Therefore, the present study aimed to analyse the Spanish *LaLiga* teams’ performance taking some key competitive performance variables into account over a continuous period of eight seasons according to the final league ranking.

## Materials and methods

### Sample

For the aim of this study, all teams’ performances in the Spanish *LaLiga* across eight consecutive seasons (from 2011–12 to 2018–19) were analysed. All matches where the information required was not available were excluded, as well as matches where one or more players were sent off. As a result, out of a possible 6,080 performances (20 teams, each playing 38 matches throughout the eight seasons), a total of 5,518 performances were analysed, representing 90% of all the possible matches. During the eight-season period, 32 teams participated in the men’s top professional football division from Spain. All the teams were divided into four groups according to the final league ranking each season: Europe (from 1^st^ to 6^th^; n = 1,642), Upper-Middle (from 7^th^ to 11^th^; n = 1,389), Lower-Middle (from 12^th^ to 17^th^; n = 1,656) and Relegation (from 18^th^ to 20^th^; n = 831). The data to carry out this study was collected in June 2019, after the end of the 2018–2019 season.

Data were obtained from the Spanish *Professional Football League*, which authorised the use of the variables included in this investigation. Following its ethical guidelines, this investigation does not include information that identifies football players. Data were treated in accordance with the Declaration of Helsinki, having been approved by the Ethics Committee on Humans (CEISH) of the *University of the Basque Country* (UPV/EHU).

### Variables

The variables used in this work were grouped into four dimensions: Technical-Tactical (Passes, Successful Passes, Crosses and Shots), Set Piece (Goals, Corners and Fouls), Collective Tactical Behaviour (Width, Length, Height and distance from the goalkeeper to the nearest defender (GkDef)) and Physical (total distance covered (TD)). Table 1 shows the definitions of these variables for each dimension. The number of points accumulated by the Spanish *LaLiga* teams was also calculated in each of the eight seasons.

**Table 1:** Definitions of the variables for each dimension.

Dimensions	Variables	Definitions
Technical-Tactical	Passes	An intentional played ball from one player to another with any part of the body that is allowed in the rules of the game. When calculating this variable, the total number of successful and unsuccessful actions made by the team per match are considered.
	Successful Passes	A successful pass is one that reaches its recipient. To calculate this variable, the total number of successful exchanges of the ball between two players of the same team per match are considered.
	Crosses	Balls sent into the rival team’s penalty box from a side area of the football pitch. When calculating this variable, the total number of successful and unsuccessful actions made by the team per match are considered.
	Shots	Attempt to score a goal, made with any part of the body that is allowed in the rules of the game. When calculating this variable, the total number of actions made by the team per match are considered.
Set Piece	Goals	Total number of points scored by each team per match.
	Corners	A kick that is performed on a set piece from the corner of the football pitch nearest to where the ball went out of the playing area. When calculating this variable, the total number of actions taken by the team per match are considered.
	Fouls	Any infringement that is penalised as foul play by the referee. When calculating this variable, the total number of actions received by the team per match are considered.
Collective Tactical Behaviour	Width	Mean team amplitude per match, considered as the distance (in m) between the two furthest-apart players of the same team along the amplitude of the pitch. To calculate this variable, the times in which the ball is out of play and the goalkeeper’s activity are excluded.
	Length	Mean team depth per match, considered as the distance (in m) between the two furthest-apart players of the same team along the depth of the pitch. To calculate this variable, the times in which the ball is out of play and the goalkeeper’s activity are excluded.
	Height	Mean team defence depth per match, considered as the distance (in m) between the furthest back player and the goal line he is defending. To calculate this variable, the times in which the ball is out of play and the goalkeeper’s activity are excluded.
	GkDef	Mean distance (in m) from the goalkeeper to the nearest defender of the same team per match. To calculate this variable, the times in which the ball is out of play is excluded.
Physical	TD	Total distance covered (in m) by all the team’s players that participated in the match, including the goalkeeper’s activity.

### Procedures

Location and motion data were obtained using the computerised multi-camera tracking system *TRACAB* (*ChyronHego*, New York, USA), and events were obtained by the data company *OPTA* (*Opta Sports*, London, UK), both using *Mediacoach* software (*LaLiga*, Madrid, Spain). The reports were generated using *Mediacoach*, for the predefined performance indicators. The reliability of the *OPTA* system has been previously proved [15], and the reliability of the multi-camera tracking system *TRACAB* has also been tested for positioning and physical performance of the players [16]. The generated reports were exported into a *Microsoft Excel* spreadsheet (*Microsoft Corporation*, Washington, USA) to configure a matrix.

### Statistical analysis

The statistical analysis was conducted using the software *jamovi 2*.*4*.*8* [17] for *Windows*. A linear mixed model was carried out for each dependent variable in order to analyse the differences in teams’ match performance according to the group and season. Group and season were considered as fixed effects and team as random effect. The Akaike information criterion (AIC) [18] and a likelihood ratio test [19] were used to select the model that best fitted each variable. The maximum likelihood (ML) estimation was used for model comparison and for the final model of each variable the best model again using restricted maximum likelihood (REML) estimation was refitted [19]. Marginal and conditional R^2^ metrics [20] were provided for each linear mixed model as a measure of effect sizes. Marginal R^2^ is concerned with variance explained by fixed effects, and conditional R^2^ is concerned with variance explained by both fixed and random effects [20]. The level of significance was set at p<0.05.

## Results

Table 2 shows the effects of season for each group and the effects of group on the variables of the Technical-Tactical dimension. In the Europe group, the teams showed fewer Crosses in 2017–18 (-6.309; p = 0.008) and 2018–19 (-4.559; p = 0.051) compared to the 2011–12 season. In the Upper-Middle, the teams showed fewer Crosses in 2018–19 (-4.835; p = 0.050) compared to the 2011–12 season. In the Lower-Middle, the teams showed fewer Crosses in 2016–17 (-3.563; p = 0.048) compared to the 2011–12 season, and fewer Shots in 2013–14 (-1.646; p = 0.006), 2014–15 (-2.044; p<0.001), 2015–16 (-2.128; p<0.001), 2016–17 (-1.432; p = 0.015) and 2017–18 (-1.792; p = 0.003) compared to the 2011–12 season. In the Relegation group, the teams showed more Crosses in 2013–14 (5.675; p = 0.008) compared to the 2011–12 season. Likewise, Europe showed more Passes than Upper-Middle (79.536; p<0.001), Lower-Middle (94.736; p<0.001) and Relegation (112.009; p<0.001), more Successful Passes than Upper-Middle (90.069; p<0.001), Lower-Middle (103.856; p<0.001) and Relegation (119.975; p<0.001), and more Shots than Upper-Middle (2.030; p<0.001), Lower-Middle (2.541; p<0.001) and Relegation (2.779; p<0.001) during the whole period analysed.

**Table 2:** Effects of season for each group and effects of group on the variables of the Technical-Tactical dimension.

* *			**Passes**										**Successful Passes**									**Crosses**									**Shots**								
Europe	**Fixed Effects**		**Estimate**		**SE**					**p**			**Estimate**	**SE**					**p**			**Estimate**			**SE**			**p**			**Estimate**			**SE**			**p**		
	Intercept		538.655		15.464					<0.001			431.582	16.736					<0.001			19.401			0.569			<0.001			14.072			0.416			<0.001		
	2012–13–2011–12		0.272		61.799					0.997			3.004	66.889					0.964			0.016			2.267			0.994			-0.406			1.657			0.808		
	2013–14–2011–12		-21.646		62.008					0.729			-16.619	67.084					0.806			-0.663			2.304			0.775			-0.719			1.676			0.670		
	2014–15–2011–12		-25.669		61.811					0.680			-21.100	66.901					0.754			-0.061			2.270			0.979			-1.206			1.659			0.471		
	2015–16–2011–12		5.243		61.802					0.933			6.999	66.892					0.917			-3.567			2.268			0.124			-1.570			1.658			0.349		
	2016–17–2011–12		14.796		61.796					0.812			24.839	66.886					0.712			-4.202			2.267			0.071			-0.822			1.657			0.623		
	2017–18–2011–12		18.224		61.845					0.770			36.547	66.932					0.588			-6.309			2.277			0.008			-1.193			1.662			0.477		
	2018–19–2011–12		-19.907		61.808					0.749			-10.282	66.898					0.879			-4.559			2.269			0.051			-1.745			1.658			0.299		
	**Random Effects**		**SD**		**Variance**					**ICC**			**SD**	**Variance**					**ICC**			**SD**			**Variance**			**ICC**			**SD**			**Variance**			**ICC**		
	Team		105.858		11,205.967					0.559			114.765	13,170.924					0.598			3.667			13.445			0.162			2.748			7.551			0.237		
	Residual		94.116		8,857.737								94.160	8,866.170								8.348			69.687						4.934			24.340					
	Marginal R^2^ / Conditional R^2^		0.012 / 0.564										0.015 / 0.604									0.062 / 0.214									0.010 / 0.244								
Upper-Middle	**Fixed Effects**		**Estimate**		**SE**					**p**			**Estimate**	**SE**					**p**			**Estimate**			**SE**			**p**			**Estimate**			**SE**			**p**		
	Intercept		459.217		9.719					<0.001			341.609	10.231					<0.001			20.644			0.593			<0.001			12.054			0.242			<0.001		
	2012–13–2011–12		-21.570		38.886					0.583			-9.211	40.930					0.823			-1.710			2.375			0.477			0.790			0.970			0.422		
	2013–14–2011–12		-10.837		38.899					0.782			7.659	40.942					0.853			-0.348			2.378			0.884			0.132			0.972			0.893		
	2014–15–2011–12		15.168		38.895					0.699			21.233	40.938					0.608			0.617			2.377			0.797			-0.383			0.971			0.696		
	2015–16–2011–12		7.179		38.853					0.855			20.329	40.900					0.623			-1.881			2.370			0.433			-0.965			0.967			0.326		
	2016–17–2011–12		-15.707		38.864					0.689			-4.711	40.911					0.909			-1.118			2.372			0.641			-0.872			0.968			0.374		
	2017–18–2011–12		-16.655		38.895					0.671			-4.112	40.938					0.921			-1.523			2.377			0.526			-0.532			0.971			0.588		
	2018–19–2011–12		15.618		38.869					0.691			41.143	40.916					0.322			-4.835			2.373			0.050			-0.771			0.968			0.432		
	**Random Effects**		**SD**		**Variance**					**ICC**			**SD**	**Variance**					**ICC**			**SD**			**Variance**			**ICC**			**SD**			**Variance**			**ICC**		
	Team		59.789		3,574.740					0.336			63.190	3,992.937					0.373			3.470			12.039			0.145			1.329			1.765			0.080		
	Residual		83.994		7,054.980								81.879	6,704.184								8.428			71.024						4.496			20.213					
	Marginal R^2^ / Conditional R^2^		0.018 / 0.348										0.024 / 0.388									0.028 / 0.169									0.014 / 0.093								
Lower-Middle		**Fixed Effects**		**Estimate**			**SE**		**p**			**Estimate**				**SE**		**p**			**Estimate**			**SE**			**p**			**Estimate**			**SE**			**p**		
		Intercept		443.980			7.579		<0.001			327.795				7.953		<0.001			19.481			0.437			<0.001			11.540			0.141			<0.001		
		2012–13–2011–12		-6.686			30.305		0.827			-8.816				31.799		0.783			0.791			1.745			0.653			-1.035			0.564			0.074		
		2013–14–2011–12		-30.866			30.351		0.315			-25.131				31.840		0.435			0.248			1.752			0.888			-1.646			0.570			0.006		
		2014–15–2011–12		-36.742			30.305		0.233			-30.793				31.799		0.339			0.669			1.745			0.704			-2.044			0.564			<0.001		
		2015–16–2011–12		-36.629			30.282		0.234			-31.578				31.779		0.326			-2.117			1.741			0.231			-2.128			0.561			<0.001		
		2016–17–2011–12		20.206			30.304		0.509			34.296				31.798		0.287			-3.563			1.745			0.048			-1.432			0.564			0.015		
		2017–18–2011–12		-8.580			30.382		0.779			6.796				31.868		0.832			-2.438			1.757			0.173			-1.792			0.573			0.003		
		2018–19–2011–12		-20.031			30.287		0.512			-7.966				31.783		0.803			-0.583			1.742			0.739			-1.038			0.561			0.072		
		**Random Effects**		**SD**			**Variance**		**ICC**			**SD**				**Variance**		**ICC**			**SD**			**Variance**			**ICC**			**SD**			**Variance**			**ICC**		
		Team		50.556			2,555.895		0.271			53.358				2,847.104		0.306			2.690			7.234			0.099			0.644			0.415			0.022		
		Residual		83.011			6,890.763					80.298				6,447.696					8.107			65.729						4.319			18.653					
		Marginal R^2^ / Conditional R^2^		0.036 / 0.297								0.045 / 0.337									0.031 / 0.127									0.022 / 0.043								
Relegation		**Fixed Effects**		**Estimate**			**SE**		**p**			**Estimate**				**SE**		**p**			**Estimate**			**SE**			**p**			**Estimate**			**SE**			**p**		
		Intercept		426.731			8.684		<0.001			311.694				9.130		<0.001			19.791			0.465			<0.001			11.304			0.217			<0.001		
		2012–13–2011–12		-7.955			34.753		0.822			-3.391				36.533		0.927			3.855			1.864			0.055			1.393			0.869			0.129		
		2013–14–2011–12		12.839			34.719		0.716			11.991				36.503		0.747			5.675			1.857			0.008			-0.120			0.865			0.892		
		2014–15–2011–12		-33.577			34.733		0.348			-32.347				36.516		0.389			2.297			1.860			0.235			-1.752			0.867			0.061		
		2015–16–2011–12		20.363			34.734		0.566			21.135				36.517		0.571			3.759			1.860			0.060			0.766			0.867			0.390		
		2016–17–2011–12		-23.200			34.715		0.514			-17.768				36.500		0.633			-0.758			1.857			0.689			-1.391			0.865			0.128		
		2017–18–2011–12		39.490			34.744		0.272			51.978				36.525		0.174			0.643			1.862			0.734			-0.022			0.868			0.980		
		2018–19–2011–12		-1.156			34.753		0.974			13.005				36.533		0.727			2.685			1.864			0.169			0.143			0.869			0.872		
		**Random Effects**		**SD**			**Variance**		**ICC**			**SD**				**Variance**		**ICC**			**SD**			**Variance**			**ICC**			**SD**			**Variance**			**ICC**		
		Team		40.113			1,609.042		0.188			42.584				1,813.360		0.219			1.789			3.201			0.044			0.749			0.561			0.028		
		Residual		83.324			6,942.861					80.406				6,465.090					8.300			68.889						4.428			19.609					
		Marginal R^2^ / Conditional R^2^		0.054 / 0.232								0.065 / 0.270									0.055 / 0.097									0.044 / 0.071								
All seasons	**Fixed Effects**		**Estimate**			**SE**		**p**			**Estimate**				**SE**		**p**			**Estimate**			**SE**			**p**			**Estimate**			**SE**			**p**		
	Intercept		467.184			5.770		<0.001			353.206				6.201		<0.001			19.826			0.302			<0.001			12.245			0.151			<0.001		
	Upper-Middle—Europe		-79.536			15.016		<0.001			-90.069				16.137		<0.001			1.250			0.785			0.114			-2.030			0.394			<0.001		
	Lower-Middle—Europe		-94.736			14.320		<0.001			-103.856				15.389		<0.001			0.089			0.749			0.905			-2.541			0.376			<0.001		
	Relegation—Europe		-112.009			17.532		<0.001			-119.975				18.842		<0.001			0.400			0.917			0.663			-2.779			0.460			<0.001		
	**Random Effects**		**SD**			**Variance**		**ICC**			**SD**				**Variance**		**ICC**			**SD**			**Variance**			**ICC**			**SD**			**Variance**			**ICC**		
	Team		68.529			4,696.183		0.384			73.938				5,466.775		0.430			3.380			11.424			0.143			1.666			2.777			0.117		
	Residual		86.747			7,524.955					85.055				7,234.320					8.289			68.715						4.570			20.883					
	Marginal R^2^ / Conditional R^2^		0.135 / 0.467								0.152 / 0.517									0.003 / 0.145									0.051 / 0.162								

Note: SE is Standard Error; SD is Standard Deviation; ICC is Intraclass Correlation Coefficient. Statistical significance set at p<0.05.

Table 3 shows the effects of season for each group and the effects of group on the variables of the Set Piece dimension. In the Europe group, the teams showed fewer Corners in 2016–17 (-1.338; p = 0.015) compared to the 2011–12 season, and fewer Fouls in 2015–16 (-1.585; p = 0.027) and 2017–18 (-1.390; p = 0.052) compared to the 2011–12 season. In the Upper-Middle, the teams showed more Goals in 2012–13 (0.314; p = 0.020) and 2016–17 (0.312; p = 0.020) compared to the 2011–12 season. In the Lower-Middle, the teams showed fewer Goals in 2013–14 (-0.323; p = 0.008) and 2014–15 (-0.368; p = 0.002) compared to the 2011–12 season, fewer Corners in 2016–17 (-0.734; p = 0.047) compared to the 2011–12 season, and fewer Fouls in 2018–19 (-1.887; p = 0.017) compared to the 2011–12 season. In the Relegation group, the teams showed more Corners in 2013–14 (1.444; p = 0.018) compared to the 2011–12 season, and fewer Fouls in 2016–17 (-3.033; p = 0.035) compared to the 2011–12 season. Likewise, Europe showed more Goals than Upper-Middle (0.820; p<0.001), Lower-Middle (0.877; p<0.001) and Relegation (1.037; p<0.001), more Corners than Upper-Middle (0.538; p = 0.004), Lower-Middle (0.837; p<0.001) and Relegation (0.859; p<0.001), and more Fouls than Relegation (0.813; p = 0.016) during the whole period analysed.

**Table 3:** Effects of season for each group and effects of group on the variables of the Set Piece dimension.

* *		**Goals**			**Corners**			**Fouls**		
Europe	**Fixed Effects**	**Estimate**	**SE**	**p**	**Estimate**	**SE**	**p**	**Estimate**	**SE**	**p**
	Intercept	2.008	0.097	<0.001	5.691	0.132	<0.001	14.326	0.174	<0.001
	2012–13–2011–12	0.007	0.385	0.985	-0.426	0.524	0.421	-0.280	0.688	0.687
	2013–14–2011–12	0.063	0.391	0.872	0.152	0.539	0.780	-0.688	0.711	0.339
	2014–15–2011–12	0.021	0.385	0.957	-0.685	0.525	0.200	-0.355	0.690	0.609
	2015–16–2011–12	-0.122	0.385	0.752	-0.650	0.524	0.223	-1.585	0.689	0.027
	2016–17–2011–12	0.041	0.385	0.915	-1.338	0.523	0.015	-0.478	0.687	0.491
	2017–18–2011–12	-0.043	0.387	0.913	-1.039	0.529	0.057	-1.390	0.696	0.052
	2018–19–2011–12	-0.427	0.385	0.274	-0.876	0.525	0.103	-1.189	0.690	0.092
	**Random Effects**	**SD**	**Variance**	**ICC**	**SD**	**Variance**	**ICC**	**SD**	**Variance**	**ICC**
	Team	0.618	0.382	0.148	0.757	0.573	0.061	0.960	0.921	0.050
	Residual	1.481	2.193		2.971	8.829		4.197	17.616	
	Marginal R^2^ / Conditional R^2^	0.009 / 0.156			0.022 / 0.082			0.016 / 0.065		
Upper-Middle	**Fixed Effects**	**Estimate**	**SE**	**p**	**Estimate**	**SE**	**p**	**Estimate**	**SE**	**p**
	Intercept	1.190	0.032	<0.001	5.146	0.141	<0.001	14.132	0.183	<0.001
	2012–13–2011–12	0.314	0.128	0.020	-0.012	0.566	0.984	0.118	0.732	0.873
	2013–14–2011–12	0.189	0.129	0.152	-0.279	0.567	0.626	0.100	0.734	0.892
	2014–15–2011–12	0.143	0.129	0.276	-0.007	0.567	0.990	0.412	0.733	0.578
	2015–16–2011–12	0.097	0.126	0.448	-0.440	0.564	0.441	0.676	0.728	0.360
	2016–17–2011–12	0.312	0.127	0.020	-0.754	0.564	0.191	-0.821	0.729	0.269
	2017–18–2011–12	0.118	0.129	0.365	-0.327	0.567	0.568	-0.330	0.733	0.656
	2018–19–2011–12	0.157	0.127	0.226	-1.113	0.565	0.057	0.402	0.730	0.586
	**Random Effects**	**SD**	**Variance**	**ICC**	**SD**	**Variance**	**ICC**	**SD**	**Variance**	**ICC**
	Team	0.070	0.005	0.004	0.766	0.587	0.074	0.915	0.838	0.046
	Residual	1.115	1.243		2.711	7.350		4.151	17.228	
	Marginal R^2^ / Conditional R^2^	0.008 / 0.012			0.017 / 0.090			0.011 / 0.057		
Lower-Middle	**Fixed Effects**	**Estimate**	**SE**	**p**	**Estimate**	**SE**	**p**	**Estimate**	**SE**	**p**
	Intercept	1.131	0.028	<0.001	4.847	0.090	<0.001	13.862	0.190	<0.001
	2012–13–2011–12	-0.221	0.113	0.059	0.107	0.358	0.766	-0.925	0.758	0.229
	2013–14–2011–12	-0.323	0.115	0.008	-0.000	0.361	0.999	-0.527	0.763	0.494
	2014–15–2011–12	-0.368	0.113	0.002	-0.365	0.358	0.314	-0.696	0.758	0.364
	2015–16–2011–12	-0.117	0.112	0.302	-0.620	0.356	0.089	-1.258	0.756	0.104
	2016–17–2011–12	-0.034	0.113	0.766	-0.734	0.358	0.047	0.577	0.758	0.451
	2017–18–2011–12	-0.092	0.116	0.434	-0.595	0.363	0.109	-0.633	0.765	0.412
	2018–19–2011–12	-0.093	0.112	0.415	-0.460	0.356	0.204	-1.887	0.757	0.017
	**Random Effects**	**SD**	**Variance**	**ICC**	**SD**	**Variance**	**ICC**	**SD**	**Variance**	**ICC**
	Team	0.062	0.004	0.003	0.429	0.184	0.026	1.123	1.261	0.073
	Residual	1.096	1.200		2.631	6.921		4.007	16.058	
	Marginal R^2^ / Conditional R^2^	0.013 / 0.016			0.013 / 0.038			0.028 / 0.099		
Relegation	**Fixed Effects**	**Estimate**	**SE**	**p**	**Estimate**	**SE**	**p**	**Estimate**	**SE**	**p**
	Intercept	0.973	0.036	<0.001	4.822	0.137	<0.001	13.518	0.329	<0.001
	2012–13–2011–12	0.187	0.144	0.213	0.744	0.549	0.194	-1.099	1.318	0.416
	2013–14–2011–12	-0.007	0.143	0.961	1.444	0.547	0.018	-2.555	1.316	0.070
	2014–15–2011–12	-0.115	0.143	0.436	-0.338	0.548	0.546	-2.003	1.317	0.148
	2015–16–2011–12	0.175	0.143	0.240	0.440	0.548	0.434	-2.240	1.317	0.108
	2016–17–2011–12	0.021	0.143	0.886	-0.528	0.547	0.349	-3.033	1.315	0.035
	2017–18–2011–12	-0.146	0.144	0.325	0.105	0.549	0.851	-1.126	1.317	0.405
	2018–19–2011–12	0.137	0.144	0.356	-0.286	0.549	0.609	-2.237	1.318	0.109
	**Random Effects**	**SD**	**Variance**	**ICC**	**SD**	**Variance**	**ICC**	**SD**	**Variance**	**ICC**
	Team	0.069	0.005	0.005	0.512	0.262	0.039	1.447	2.094	0.107
	Residual	0.950	0.902		2.552	6.512		4.186	17.526	
	Marginal R^2^ / Conditional R^2^	0.015 / 0.020			0.053 / 0.090			0.041 / 0.144		
All seasons	**Fixed Effects**	**Estimate**	**SE**	**p**	**Estimate**	**SE**	**p**	**Estimate**	**SE**	**p**
	Intercept	1.325	0.032	<0.001	5.123	0.070	<0.001	13.959	0.110	<0.001
	Upper-Middle—Europe	-0.820	0.084	<0.001	-0.538	0.181	0.004	-0.196	0.285	0.494
	Lower-Middle—Europe	-0.877	0.080	<0.001	-0.837	0.173	<0.001	-0.469	0.272	0.087
	Relegation—Europe	-1.037	0.098	<0.001	-0.859	0.212	<0.001	-0.813	0.333	0.016
	**Random Effects**	**SD**	**Variance**	**ICC**	**SD**	**Variance**	**ICC**	**SD**	**Variance**	**ICC**
	Team	0.331	0.109	0.070	0.705	0.497	0.062	1.131	1.278	0.070
	Residual	1.209	1.462		2.745	7.534		4.128	17.037	
	Marginal R^2^ / Conditional R^2^	0.098 / 0.161			0.016 / 0.077			0.004 / 0.074		

Note: SE is Standard Error; SD is Standard Deviation; ICC is Intraclass Correlation Coefficient. Statistical significance set at p<0.05.

Table 4 shows the effects of season for each group and the effects of group on the variables of the Collective Tactical Behaviour dimension. In the Europe group, the teams showed lower values of Length in 2015–16 (-1.665; p = 0.015), 2016–17 (-1.613; p = 0.019), 2017–18 (-1.930; p = 0.006) and 2018–19 (-2.276; p = 0.001) compared to the season 2011–12, and lower values of GkDef in 2014–15 (-3.190; p = 0.001), 2015–16 (-3.169; p = 0.001), 2016–17 (-2.722; p = 0.005), 2017–18 (-2.633; p = 0.007) and 2018–19 (-2.487; p = 0.010) compared to the season 2011–12. In the Upper-Middle group, the teams showed lower values of Length in 2015–16 (-1.622; p = 0.001), 2016–17 (-2.706; p<0.001), 2017–18 (-2.463; p<0.001) and 2018–19 (-1.952; p<0.001) compared to the season 2011–12. In the Lower-Middle group, the teams showed lower values of Length in 2014–15 (-1.218; p = 0.040), 2015–16 (-1.660; p = 0.006), 2016–17 (-1.609; p = 0.008), 2017–18 (-2.211; p<0.001) and 2018–19 (-2.542; p<0.001) compared to the season 2011–12, lower values of Height in 2014–15 (-1.407; p = 0.040) compared to the season 2011–12, and lower values of GkDef in 2014–15 (-2.002; p<0.001), 2015–16 (-1.668; p = 0.002), 2016–17 (-1.839; p<0.001), 2017–18 (-1.747; p = 0.001) and 2018–19 (-1.371; p = 0.009) compared to the season 2011–12. In the Relegation group, the teams showed lower values of Length in 2016–17 (-1.851; p = 0.006) and 2018–19 (-1.263; p = 0.044) compared to the 2011–12 season, lower values of Height in 2014–15 (-1.893; p = 0.043) compared to the 2011–12 season, and lower values of GkDef in 2014–15 (-3.638; p<0.001) and 2015–16 (-2.506; p = 0.009) compared to the 2011–12 season. Likewise, Europe showed higher values of Width than Upper-Middle (0.928; p = 0.009), Lower-Middle (1.010; p = 0.003) and Relegation (1.373; p = 0.001), higher values of Length than Upper-Middle (0.667; p = 0.010), Lower-Middle (0.756; p = 0.002) and Relegation (1.055; p<0.001), higher values of Height than Upper-Middle (1.164; p<0.001), Lower-Middle (1.412; p<0.001) and Relegation (1.726; p<0.001), and higher values of GkDef than Upper-Middle (1.175; p<0.001), Lower-Middle (0.985; p = 0.002) and Relegation (0.871; p = 0.026) during the whole period analysed.

**Table 4:** Effects of season for each group and effects of group on the variables of the Collective Tactical Behaviour dimension.

* *		**Width**			**Length**			**Height**			**GkDef**		
Europe	**Fixed Effects**	**Estimate**	**SE**	**p**	**Estimate**	**SE**	**p**	**Estimate**	**SE**	**p**	**Estimate**	**SE**	**p**
	Intercept	44.250	0.321	<0.001	37.475	0.165	<0.001	38.402	0.313	<0.001	25.730	0.230	<0.001
	2012–13–2011–12	0.335	1.283	0.795	-0.285	0.657	0.667	-0.311	1.251	0.805	0.326	0.919	0.724
	2013–14–2011–12	-0.219	1.288	0.866	-0.716	0.662	0.286	-0.509	1.262	0.688	-0.718	0.924	0.442
	2014–15–2011–12	-0.679	1.284	0.600	-1.056	0.658	0.116	-2.192	1.251	0.087	-3.190	0.919	0.001
	2015–16–2011–12	-0.218	1.283	0.866	-1.665	0.657	0.015	-1.411	1.251	0.266	-3.169	0.919	0.001
	2016–17–2011–12	0.892	1.283	0.491	-1.613	0.657	0.019	-1.277	1.250	0.313	-2.722	0.919	0.005
	2017–18–2011–12	0.509	1.284	0.694	-1.930	0.658	0.006	-2.043	1.253	0.111	-2.633	0.920	0.007
	2018–19–2011–12	-0.599	1.284	0.643	-2.276	0.658	0.001	-1.369	1.251	0.280	-2.487	0.919	0.010
	**Random Effects**	**SD**	**Variance**	**ICC**	**SD**	**Variance**	**ICC**	**SD**	**Variance**	**ICC**	**SD**	**Variance**	**ICC**
	Team	2.199	4.834	0.561	1.110	1.232	0.354	2.098	4.401	0.301	1.560	2.433	0.411
	Residual	1.944	3.780		1.499	2.246		3.199	10.233		1.866	3.483	
	Marginal R^2^ / Conditional R^2^	0.030 / 0.574			0.143 / 0.447			0.036 / 0.326			0.241 / 0.553		
Upper-Middle	**Fixed Effects**	**Estimate**	**SE**	**p**	**Estimate**	**SE**	**p**	**Estimate**	**SE**	**p**	**Estimate**	**SE**	**p**
	Intercept	43.324	0.263	<0.001	36.807	0.116	<0.001	37.234	0.266	<0.001	24.552	0.201	<0.001
	2012–13–2011–12	0.376	1.051	0.723	-0.196	0.463	0.675	-0.122	1.065	0.909	0.120	0.802	0.882
	2013–14–2011–12	0.729	1.051	0.493	-0.737	0.463	0.121	0.045	1.066	0.966	0.692	0.802	0.395
	2014–15–2011–12	-0.086	1.051	0.935	-0.786	0.463	0.099	-0.110	1.066	0.918	-0.944	0.802	0.248
	2015–16–2011–12	0.171	1.050	0.871	-1.622	0.462	0.001	-0.170	1.064	0.874	-0.881	0.802	0.280
	2016–17–2011–12	-0.518	1.050	0.625	-2.706	0.462	<0.001	0.352	1.065	0.743	-0.691	0.802	0.395
	2017–18–2011–12	-0.075	1.051	0.943	-2.463	0.463	<0.001	0.392	1.066	0.716	-0.240	0.802	0.767
	2018–19–2011–12	0.479	1.050	0.651	-1.952	0.462	<0.001	-0.066	1.065	0.951	-1.341	0.802	0.104
	**Random Effects**	**SD**	**Variance**	**ICC**	**SD**	**Variance**	**ICC**	**SD**	**Variance**	**ICC**	**SD**	**Variance**	**ICC**
	Team	1.623	2.633	0.374	0.674	0.454	0.141	1.611	2.594	0.236	1.237	1.531	0.364
	Residual	2.099	4.404		1.665	2.771		2.896	8.386		1.636	2.677	
	Marginal R^2^ / Conditional R^2^	0.019 / 0.386			0.222 / 0.331			0.004 / 0.239			0.085 / 0.418		
Lower-Middle	**Fixed Effects**	**Estimate**	**SE**	**p**	**Estimate**	**SE**	**p**	**Estimate**	**SE**	**p**	**Estimate**	**SE**	**p**
	Intercept	43.241	0.206	<0.001	36.718	0.144	<0.001	36.987	0.166	<0.001	24.744	0.126	<0.001
	2012–13–2011–12	-1.264	0.825	0.133	-0.914	0.574	0.119	0.048	0.661	0.943	-0.564	0.502	0.268
	2013–14–2011–12	-0.362	0.826	0.664	-0.855	0.575	0.145	-0.085	0.664	0.898	-0.105	0.503	0.836
	2014–15–2011–12	-1.187	0.825	0.158	-1.218	0.574	0.040	-1.407	0.661	0.040	-2.002	0.502	<0.001
	2015–16–2011–12	-0.621	0.824	0.456	-1.660	0.573	0.006	-0.262	0.660	0.694	-1.668	0.502	0.002
	2016–17–2011–12	0.413	0.825	0.619	-1.609	0.574	0.008	-0.211	0.661	0.751	-1.839	0.502	<0.001
	2017–18–2011–12	-0.468	0.827	0.574	-2.211	0.576	<0.001	-0.408	0.666	0.543	-1.747	0.504	0.001
	2018–19–2011–12	-0.951	0.824	0.256	-2.542	0.573	<0.001	-0.056	0.660	0.932	-1.371	0.502	0.009
	**Random Effects**	**SD**	**Variance**	**ICC**	**SD**	**Variance**	**ICC**	**SD**	**Variance**	**ICC**	**SD**	**Variance**	**ICC**
	Team	1.382	1.911	0.298	0.949	0.900	0.229	1.036	1.073	0.115	0.824	0.680	0.203
	Residual	2.123	4.507		1.741	3.032		2.879	8.291		1.632	2.664	
	Marginal R^2^ / Conditional R^2^	0.044 / 0.329			0.127 / 0.327			0.021 / 0.133			0.146 / 0.319		
Relegation	**Fixed Effects**	**Estimate**	**SE**	**p**	**Estimate**	**SE**	**p**	**Estimate**	**SE**	**p**	**Estimate**	**SE**	**p**
	Intercept	42.877	0.212	<0.001	36.419	0.145	<0.001	36.672	0.216	<0.001	24.856	0.212	<0.001
	2012–13–2011–12	-0.574	0.848	0.508	-0.042	0.579	0.943	1.395	0.863	0.125	0.737	0.847	0.397
	2013–14–2011–12	0.127	0.848	0.883	0.159	0.578	0.787	0.427	0.861	0.627	-0.436	0.847	0.613
	2014–15–2011–12	-0.544	0.848	0.530	-0.114	0.579	0.846	-1.893	0.862	0.043	-3.638	0.847	<0.001
	2015–16–2011–12	-0.077	0.848	0.928	-0.943	0.579	0.123	0.105	0.862	0.904	-2.506	0.847	0.009
	2016–17–2011–12	-0.284	0.848	0.742	-1.851	0.578	0.006	0.469	0.861	0.593	-1.662	0.846	0.067
	2017–18–2011–12	0.648	0.848	0.456	-0.636	0.579	0.288	0.461	0.863	0.600	-1.266	0.847	0.154
	2018–19–2011–12	0.707	0.848	0.417	-1.263	0.579	0.044	-0.519	0.863	0.556	-1.569	0.847	0.083
	**Random Effects**	**SD**	**Variance**	**ICC**	**SD**	**Variance**	**ICC**	**SD**	**Variance**	**ICC**	**SD**	**Variance**	**ICC**
	Team	0.979	0.958	0.187	0.641	0.411	0.115	0.939	0.881	0.098	0.996	0.992	0.255
	Residual	2.041	4.167		1.780	3.169		2.846	8.099		1.702	2.895	
	Marginal R^2^ / Conditional R^2^	0.038 / 0.218			0.113 / 0.215			0.082 / 0.172			0.306 / 0.483		
All seasons	**Fixed Effects**	**Estimate**	**SE**	**p**	**Estimate**	**SE**	**p**	**Estimate**	**SE**	**p**	**Estimate**	**SE**	**p**
	Intercept	43.423	0.135	<0.001	36.854	0.098	<0.001	37.323	0.133	<0.001	24.969	0.127	<0.001
	Upper-Middle—Europe	-0.928	0.352	0.009	-0.667	0.254	0.010	-1.164	0.346	<0.001	-1.175	0.331	<0.001
	Lower-Middle—Europe	-1.010	0.336	0.003	-0.756	0.242	0.002	-1.412	0.330	<0.001	-0.985	0.316	0.002
	Relegation—Europe	-1.373	0.411	0.001	-1.055	0.297	<0.001	-1.726	0.404	<0.001	-0.871	0.387	0.026
	**Random Effects**	**SD**	**Variance**	**ICC**	**SD**	**Variance**	**ICC**	**SD**	**Variance**	**ICC**	**SD**	**Variance**	**ICC**
	Team	1.607	2.582	0.380	1.153	1.329	0.326	1.531	2.345	0.209	1.520	2.309	0.439
	Residual	2.053	4.214		1.659	2.753		2.977	8.864		1.716	2.946	
	Marginal R^2^ / Conditional R^2^	0.036 / 0.402			0.034 / 0.349			0.037 / 0.239			0.042 / 0.463		

Note: SE is Standard Error; SD is Standard Deviation; ICC is Intraclass Correlation Coefficient. Statistical significance set at p<0.05.

Table 5 shows the effects of season for each group and the effects of group on the variable of the Physical dimension. In the Europe group, the teams showed lower values of TD in 2018–19 (-3,200.245; p = 0.050) compared to the 2011–12 season. In the Lower-Middle group, the teams showed lower values of TD in 2014–15 (-3,741.391; p = 0.011), 2015–16 (-3,278.483; p = 0.024), 2016–17 (-3,793.554; p = 0.010) and 2018–19 (-2,863.177; p = 0.047) compared to the 2011–12 season. In the Relegation group, the teams showed lower values of TD in 2014–15 (-4,244.181; p = 0.033) compared to the 2011–12 season. Likewise, Europe showed lower values of TD than Upper-Middle (-1,527.654; p = 0.007) during the whole period analysed.

**Table 5:** Effects of season for each group and effects of group on the variable of the Physical dimension.

		**TD**		
Europe	**Fixed Effects**	**Estimate**	**SE**	**p**
	Intercept	109,316.536	395.921	<0.001
	2012–13–2011–12	-664.549	1,580.860	0.676
	2013–14–2011–12	-240.763	1,591.088	0.880
	2014–15–2011–12	-2,306.592	1,581.493	0.153
	2015–16–2011–12	-1,503.857	1,581.014	0.347
	2016–17–2011–12	-1,196.475	1,580.702	0.454
	2017–18–2011–12	-1,728.212	1,583.236	0.282
	2018–19–2011–12	-3,200.245	1,581.420	0.050
	**Random Effects**	**SD**	**Variance**	**ICC**
	Team	2,677.165	7,167,211.081	0.381
	Residual	3,411.303	11,636,987.536	
	Marginal R^2^ / Conditional R^2^	0.050 / 0.412				
Upper-Middle	**Fixed Effects**	**Estimate**	**SE**	**p**
	Intercept	110,839.230	417.130	<0.001
	2012–13–2011–12	-899.901	1,668.878	0.593
	2013–14–2011–12	-952.604	1,669.421	0.572
	2014–15–2011–12	-2,835.551	1,669.253	0.099
	2015–16–2011–12	-2,892.991	1,667.468	0.092
	2016–17–2011–12	-2,118.098	1,667.958	0.213
	2017–18–2011–12	-2,984.391	1,669.250	0.083
	2018–19–2011–12	-3,191.599	1,668.314	0.065
	**Random Effects**	**SD**	**Variance**	**ICC**
	Team	2,566.647	6,587,675.591	0.338
	Residual	3,588.856	12,879,889.799	
	Marginal R^2^ / Conditional R^2^	0.016 / 0.379				
Lower-Middle	**Fixed Effects**	**Estimate**	**SE**	**p**
	Intercept	109,912.326	349.425	<0.001
	2012–13–2011–12	22.669	1,397.123	0.987
	2013–14–2011–12	-1,846.974	1,399.127	0.194
	2014–15–2011–12	-3,741.391	1,397.098	0.011
	2015–16–2011–12	-3,278.483	1,396.099	0.024
	2016–17–2011–12	-3,793.554	1,397.074	0.010
	2017–18–2011–12	-2,090.699	1,400.508	0.143
	2018–19–2011–12	-2,863.177	1,396.528	0.047
	**Random Effects**	**SD**	**Variance**	**ICC**
	Team	2,335.007	5,452,259.170	0.281
	Residual	3,735.540	13,954,255.658	
	Marginal R^2^ / Conditional R^2^	0.097 / 0.351				
Relegation	**Fixed Effects**	**Estimate**	**SE**	**p**
	Intercept	109,480.764	454.954	<0.001
	2012–13–2011–12	-1,185.242	1,820.500	0.524
	2013–14–2011–12	-1,144.610	1,819.133	0.538
	2014–15–2011–12	-4,244.181	1,819.716	0.033
	2015–16–2011–12	669.433	1,819.756	0.718
	2016–17–2011–12	-1,653.588	1,818.975	0.377
	2017–18–2011–12	-2,233.729	1,820.138	0.238
	2018–19–2011–12	-1,771.906	1,820.500	0.345
	**Random Effects**	**SD**	**Variance**	**ICC**
	Team	2,130.215	4,537,816.447	0.234
	Residual	3,854.597	14,857,921.804	
	Marginal R^2^ / Conditional R^2^	0.090 / 0.303				
All seasons	**Fixed Effects**	**Estimate**	**SE**	**p**
	Intercept	109,885.411	216.473	<0.001
	Upper-Middle—Europe	1,527.654	563.405	0.007
	Lower-Middle—Europe	600.019	537.319	0.266
	Relegation—Europe	169.865	657.800	0.797
	**Random Effects**	**SD**	**Variance**	**ICC**
	Team	2,556.585	6,536,128.037	0.332
	Residual	3,623.655	13,130,876.084	
	Marginal R^2^ / Conditional R^2^	0.018 / 0.344				

Note: SE is Standard Error; SD is Standard Deviation; ICC is Intraclass Correlation Coefficient. Statistical significance set at p<0.05.

**Fig 1:**
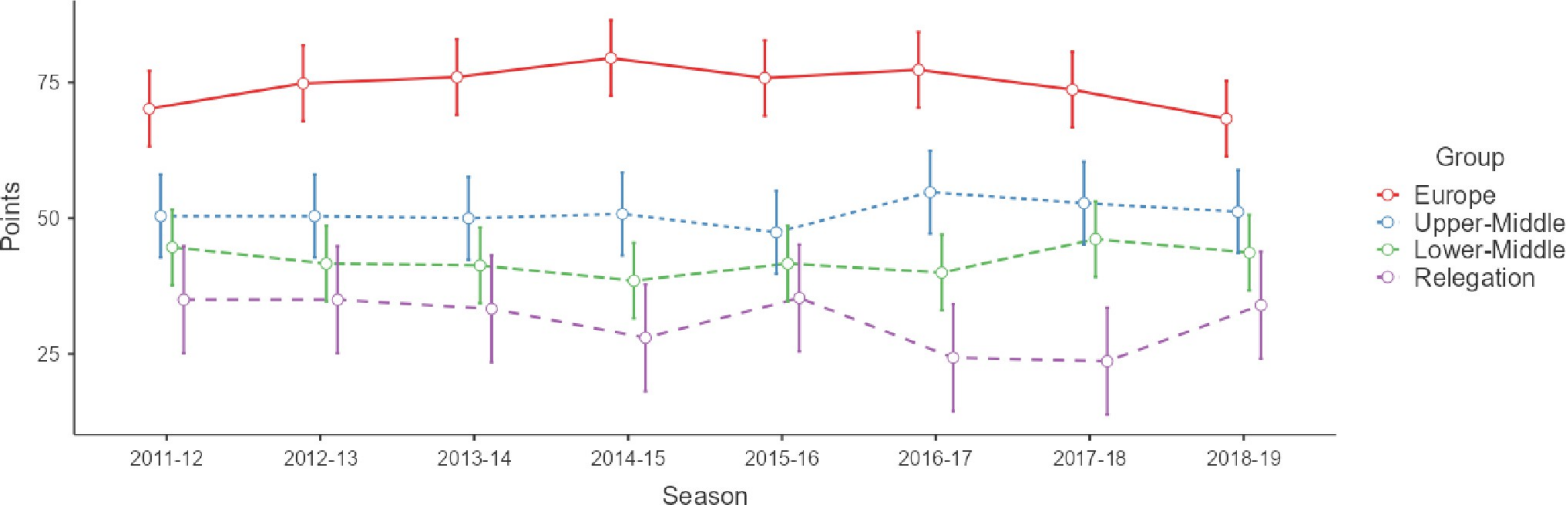
Effects of season for each group and effects of group on the number of points accumulated. Data represent the means and 95% confidence intervals.

Fig 1 shows the effects of season for each group and the effects of group on the number of points accumulated. In the Upper-Middle group, the teams showed more points in 2016–17 (5.018; p = 0.046) compared to the 2011–12 season. In the Lower-Middle group, the teams showed fewer points in 2014–15 (-5.855; p<0.001) and 2016–17 (-4.027; p = 0.022) compared to the 2011–12 season. In the Relegation group, the teams showed fewer points in 2016–17 (-10.667; p = 0.029) and 2017–18 (-11.333; p = 0.021) compared to the 2011–12 season. Likewise, Europe showed more points than Upper-Middle (13.474; p<0.001), Lower-Middle (21.795; p<0.001) and Relegation (32.177; p<0.001) during the whole period analysed.

## Discussion

The objective of this study was to analyse the performance of the Spanish *LaLiga* teams over a continuous period of eight seasons, considering the final league ranking. The main results of the study were that: 1) the Europe group showed significantly higher values compared to the other groups in most of the variables during the eight-season period; 2) the Europe group teams showed lower values of Length from the fifth season (from 2015–16 to 2018–19), and lower values of GkDef from the fourth season (from 2014–15 to 2018–19); 3) the Upper-Middle group teams showed lower values of Length from the fifth season (from 2015–16 to 2018–19); 4) the Lower-Middle group teams showed fewer Shots from the third season (from 2013–14 to 2018–19), and lower values of Length, GkDef and TD from the fourth season (from 2014–15 to 2018–19); and, 5) the Relegation group barely showed significant differences between seasons in any variable.

Regarding the Technical-Tactical dimension, the season factor had a significant effect on Crosses for Europe, Upper-Middle, Lower-Middle and Relegation, and a significant effect on Shots for Lower-Middle. The group factor also had a significant effect on Passes, Successful Passes and Shots. The distribution in these variables performed by the teams of the four groups implied greatly a performance stability throughout the analysed period. In relation to Passes and Successful Passes, the results of this work are similar to those of a recently published study about the evolution of physical and technical parameters in the Spanish *LaLiga* between the 2012–13 and 2019–20 seasons [12]. These researchers found no clear trend in the total passes as seasons progressed for any of the four groups but did find an upward trend in passing accuracy for the Top (ranked from 1^st^ to 5^th^) and Lower-Middle (ranked from 11^th^ to 15^th^) teams. However, the effect size of the differences between seasons was small. Therefore, it is worth mentioning that the study by Lago-Peñas et al. [12] also showed stability in the passes made during the analysed seasons. Bradley et al. [9], for their part, observed an increase in passes and successful passes made by the teams in the English *Premier League* over seven seasons (from 2006–07 to 2012–13). Tier A (teams ranked from 1^st^ to 4^th^) and Tier C (teams ranked from 9^th^ to 14^th^) teams significantly increased passes and successful passes made with a small effect size, Tier D (teams ranked from 15^th^ to 20^th^) teams with a moderate effect size and Tier B (teams ranked from 5^th^ to 8^th^) teams with a large effect size. A possible explanation for this could be that the teams located at the top of the ranking have been able to maintain a high and stable performance over the years, far from the more unstable performance of the rest of the teams located at the bottom of the ranking, whose annual objective is usually the one to maintain the category season after season. Another possible explanation could be that the technical-tactical dimension prevailed over the physical dimension throughout the seasons in the English *Premier League*. However, the results of the present work differ from those obtained by Bradley et al. [9].

With regard to the Crosses, it should be noted that the Europe teams showed fewer actions of this performance indicator in 2017–18 and 2018–19 compared to the 2011–12 season. Nevertheless, just like for the other three groups, the trend of Crosses over the eight seasons was quite stable for the Europe group. In the case of Shots, significant differences between seasons were only found for the Lower-Middle group. The teams of this group showed fewer Shots from the 2013–14 season. Lago-Peñas et al. [12], for their part, observed a significant decrease in the 2019–20 season compared to the 2012–13 season for the Top (from 1^st^ to 5^th^) and Upper-Middle (from 6^th^ to 10^th^) teams of the Spanish *LaLiga*. However, the effect size of these differences was small, and no trend was observed for any group as years passed. Therefore, the trend of the shots in the work of Lago-Peñas et al. [12] was quite stable throughout the period studied. When comparing the Technical-Tactical variables between groups throughout the period studied (the eight seasons together), Europe group obtained significantly higher values than the other three groups in Passes, Successful Passes and Shots. It seems that the frequency and effectiveness of shots and passes are some of the performance indicators that differentiate the most successful teams from the rest [21]. According to some works [22, 23], a high ball possession and, therefore, a high number of accumulated passes seem to be of great importance in the victory of football teams. In addition, a study that aimed to identify the statistics of the matches that best explain the success of football in the Spanish *LaLiga* using eight seasons as a sample (from 2010–11 to 2017–18), concluded that the two variables that best determine the success of a team are the effectiveness of the shots and the total number of shots made [24]. Therefore, the Europe group stood out for showing high values in the variables of the Technical-Tactical dimension that are most related to success.

With regard to the Set Piece dimension, the season factor had a significant effect on Corners and Fouls for Europe and Relegation, a significant effect on Goals for Upper-Middle, and a significant effect on Goals, Corners and Fouls for Lower-Middle. The group factor also had a significant effect on Goals, Corners and Fouls. The distribution in these variables performed by the teams of the four groups also represents a performance stability throughout the analysed period. It is worth noting that the Lower-Middle teams showed fewer Goals in 2013–14 and 2014–15 compared to the 2011–12 season. In these two seasons the teams of this group, in addition to showing fewer Shots, they showed less effectiveness in front of the rival goal. However, the trend of Goals over the eight seasons was quite stable for Lower-Middle. When comparing the Set Piece variables between groups throughout the period studied, the Europe group showed significantly higher values than the other three groups in Goals and Corners. The key factor that can determine the result in a football match, and therefore the success of a team, is the goal. Castellano [25] found that the goals scored had a very high relationship with the achievement of a greater number of points at the end of the league competition in the Spanish *LaLiga* in the 2013–14 and 2014–15 seasons. It should also be noted that corner is a performance indicator related to attacking actions that, after the effectiveness of the shots and the total number of shots taken, can best determine the success of a team, since the action occurs near the rival goal [24]. A characteristic of the best-ranked teams in a league is that they often tend to get more set pieces such as corners after maintaining high ball possession [25], especially when possession occurs in the last third of the field, close to the opponent’s goal [26]. Consequently, the success of the teams in the Europe group could be due to the fact that they also stood out for showing high values in variables that best explain the success of a team such as the goal and corner.

Regarding the Collective Tactical Behaviour dimension, the season factor had a significant effect on Length and GkDef for Europe, a significant effect on Length for Upper-Middle, and a significant effect on Length, Height and GkDef for Lower-Middle and Relegation. The group factor also had a significant effect on Width, Length, Height and GkDef. A significant decrease in Length values was found from the 2015–16 season for the Europe and Upper-Middle groups, and from the 2014–15 season for the Lower-Middle group. It seems that the teams of these groups increased the density of the effective playing space (same players in less space) as the seasons progressed. Furthermore, a significant decrease was found in GkDef values from the 2014–15 season for the Europe and Lower-Middle groups. This could be explained by the fact that the goalkeepers of these groups’ teams are demanded to play a greater role in the offensive phase of the game, requiring his participation in initiating or continuing an attack with the players closest to him, such as with his centre-backs [10]. It could also be that these teams have been able to adopt a more defensive style of play due to less ball possession during matches. For its part, Relegation group showed a stable trend in this dimension over the eight seasons. Probably low values in the Collective Tactical Behaviour variables, represented in this group with low performance [25], may be one of the reasons that justify the stability in the collective behaviour described. When comparing the Collective Tactical Behaviour variables between groups throughout the period studied, the Europe group showed significantly higher values than the other groups in Width, Length, Height and GkDef. According to a previous study [25], a greater width, length and height of the defence was associated with the teams that accumulated the highest number of points at the end of the season in the Spanish *LaLiga* (in the 2013–14 and 2014–15 seasons). It seems, therefore, that the playing style of the most successful teams (e.g., higher positions in the final ranking) have higher values in the variables that represent the collective use of space as a trait.

In relation to the Physical dimension, the season factor had a significant effect on TD for Europe, Lower-Middle and Relegation. The group factor also had a significant effect on TD. Lower-Middle showed lower values of TD from the 2014–15 season. The teams in this group probably changed the way they played over the seasons, deploying lower total distance covered. However, the teams of the other three groups showed a stability in the total distance covered throughout the eight seasons. Lago-Peñas et al. [12] found a significant decrease in the total distance covered for different groups (Top, Upper-Middle, Lower-Middle and Lower) of the Spanish *LaLiga* over the eight seasons analysed (from 2012–13 to 2019–20). When comparing the Physical variable between groups throughout the period studied, Upper-Middle was the group that obtained the highest values in this physical variable, but it only showed significantly higher values than the Europe group. It is worth mentioning that some authors [27] indicate that performance indicators of a technical-tactical nature have a greater influence than those of a conditional nature when determining the difference between the most successful teams in the championship. This is in line with the results presented by Castellano [25], who found that the total distance covered is not related to the success achieved by the teams (in this case of the Spanish men’s top and second professional football division) at the end of the championship.

The trend in the number of points accumulated by the teams in the different groups of the Spanish *LaLiga* from 2011–12 to 2018–19 was stable. English authors [9] ensured that the teams in Tier A (from 1^st^ to 4^th^) and Tier C (from 9^th^ to 14th) groups of the *Premier League* accumulated, on average, 0.43 and 0.31 fewer points season after season (from 2006–07 to 2012–13), respectively, and for their part, the teams in Tier B (from 5^th^ to 8^th^) and Tier D (from 15^th^ to 20^th^) groups 0.32 and 0.20 more points, respectively. It seems that, throughout the seven seasons analysed by these researchers, the English teams in the Tier B group (from 5^th^ to 8^th^) were closing the points gap with those that qualified for European competitions. However, this point difference between the English teams’ season after season was minimal, so it is worth mentioning that the trend in the number of points accumulated in the English *Premier League* was also stable.

The main conclusion of the study is that the teams of the Europe, Upper-Middle and Relegation groups showed a quite stable performance, while the teams of the Lower-Middle group presented a worsening in different dimensions throughout the eight seasons analysed. It could be said that the Spanish football is in a plateau period in the performance of the best teams, which showed the ability to play in spaces with high player density as the seasons passed. Furthermore, they showed higher values in variables associated with success such as Passes, Success Passes, Shots and Corners, and in variables representative of the collective use of space (Width, Length, Height and GkDef) during the whole period studied. However, this does not detract from the fact that the teams that qualify in the less good half try to propose strategies that allow them in some cases to stay in the category, playing with the goalkeepers closer and closer to their defensive line. The information provided in the present study makes it possible to have reference values that have characterized the performance of the teams for each group.

The information provided in this study, especially due to the inclusion of a large volume of performances by the Spanish *LaLiga* teams (n = 5,518) over eight seasons, makes it possible to have reference values that have characterised the performance of the teams in the dimensions and variables studied based on league ranking at the end of each season. In addition, to the authors’ knowledge, this is the first work to analyse the evolution of variables of the collective dimension (e.g., Width, Length, Height and GkDef) according to the final classification of the teams in a top-level football league over world level such as the Spanish *LaLiga*. However, the present study is not without limitations. Firstly, the performance of the teams was calculated using the means of the variables predefined by *Mediacoach*, without having the option of the authors’ obtaining different variables by calculating them by accessing the raw data. Secondly, ball possessions were not considered in this study. The physical [28] and tactical [29] responses of the teams differ when the team has possession of the ball or not. This subject, distinguishing the attack and defence phase, is suggested for future research. Thirdly, the inclusion of other technical-tactical and physical variables (e.g., recoveries, duels, types of passes, accumulated distance at high-speed, number of accumulated sprints, etc.) and contextual variables such as the change of coach, the period of the season, playing at home or away or the level of the opponent [30–32], among others, could help refine possible inferences about the performance of the teams and to better explain their variability and stability over the years. Therefore, future studies should consider different technical-tactical and physical variables and different contextual variables. Finally, it should be noted that despite the fact that eight seasons in a national league (Spanish *LaLiga*) were studied in this study, caution must be taken when extrapolating these league results to other countries or competitions with different characteristics [33]. Nevertheless, proposing this type of studies in other leagues or countries could help to better understand the evolution of the game on a more global level.
