# Supplementary material for: An eight-season analysis of the teams’ performance in the Spanish LaLiga according to the final league ranking
Source: PLoS One. 2024 Feb 28;19(2):e0299242. doi: 10.1371/journal.pone.0299242 (PMC10901331; doi:10.1371/journal.pone.0299242)
Supplement: Attachment — Submitted filename: Response to Reviewers.pdf [file pone.0299242.s001.pdf]

Dear Jovan Gardasevic,

Please find enclosed the resubmission of our article entitled “An eight-season analysis of the teams' performance in the Spanish LaLiga according to the final league ranking” (manuscript ID PONE-D-23-35487). We thank a lot the reviewers’ comments. We have answered the points raised by the Reviewer #2 and we feel they have allowed us to improve our work substantially. All the changes included in the manuscript are with track changes.

Sincerely,

Ibai Errekagorri

- **Reviewer #1:**
    - Comment: I think the topic is interesting. The idea of finding elements that bring advantage is useful for practice. The manuscript is technically adequately prepared. The authors have my support. I recommend that the manuscript be published in its current form.
    - Answer: Thanks a lot for your words and for your recommendation to publish the article. We are very glad that you liked the work.

- **Reviewer #2:**
  - Comment: Thank you very much for giving me a chance to read the study under the title "An eight-season analysis of the teams' performance in the Spanish LaLiga according to the final league ranking" (PONE-D-23-35487). I have carefully read the manuscript and my opinion is that the manuscript has a merit to be published in your reputable journal with some minor corrections. The authors aimed to analyze the Spanish LaLiga teams' performance taking some key competitive performance variables into account over a continuous period of eight seasons according to the final league ranking. The manuscript is original, informative and readable while the study design and writing style are on the adequate level. The introduction is well written, as well as materials and methods section that is very well prepared and organized according to contemporary methodological rules. At the end, I have no amendments on results and discussion part but I would recommend to the authors to prepare the conclusion part in the following order: the main conclusions, the limitations of the study (more precisely) as well as recommendations for the further studies (it is very important to briefly elaborate it and highlight the most important notes) as this part might help some upcoming researchers to set up the monitoring systems to suit them more in the future. Lastly, I would recommend to accept this manuscript right after the authors revise it in the adequate manner.
  - Answer: Thanks a lot for your words and for your recommendation. We have modified the conclusions section according to your suggestions.
